# Supplementary material for: Correlation between Gut Microbiota and Six Facets of Neuroticism in Korean Adults
Source: J Pers Med. 2021 Nov 24;11(12):1246. doi: 10.3390/jpm11121246 (PMC8704006; doi:10.3390/jpm11121246)
Supplement: Supplementary file 1 [file jpm-11-01246-s001.zip › jpm-1476590-supplementary.pdf]

Supplementary Information Files

Correlation between gut microbiota and six facets of neuroticism in Korean adults

Eunkyo Park[1], Kyung Eun Yun[2], Mi-Hyun Kim[2], Jimin Kim[2], Yoosoo Chang[2,3,4], Seungho Ryu[2,3,4], Hyung-Lae Kim[1], Han-Na Kim[2,4,5,*], Sung-Chul Jung[1,6,*]

[1] Department of Biochemistry, College of Medicine, Ewha Womans University, Seoul, Republic of Korea; eunkyo85@gmail.com (E.P.), hyung@ewha.ac.kr (H.L.K.)
[2] Center for Cohort Studies, Total Healthcare Center, Kangbuk Samsung Hospital, Sungkyunkwan University School of Medicine, Seoul, Republic of Korea; eun3579@hanmail.net (K.Y), mh0303.kim@samsung.com (M.K.), jimin.kim@samsung.com (J.K)
[3] Department of Occupational and Environmental Medicine, Kangbuk Samsung Hospital, Sungkyunkwan University School of Medicine, Seoul, Republic of Korea; yoosoo.chang@gmail.com (Y.C.), sh703.yoo@gmail.com (S.R.)
[4] Department of Clinical Research Design and Evaluation, SAIHST, Sungkyunkwan University, Seoul, Republic of Korea. Occupational and Environmental Medicine, Kangbuk Samsung Hospital, Sungkyunkwan University School of Medicine, Seoul, Republic of Korea.
[5] Medical Research Institute, Kangbuk Samsung Hospital, Sungkyunkwan University School of Medicine, Seoul, Republic of Korea
[6] Graduate Program in System Health Science and Engineering, Ewha Womans University, Seoul, Republic of Korea
* Correspondence: jungsc@ewha.ac.kr (S.J.) & 147942@hanmail.net (H.N.K.)

Supplementary Table S1. Statistical significance between the low and high groups using distance matrices for beta-diversity.

		pseudo-F	p-value
N<br>Neuroticism	Jaccard	1.135	0.106
	Bray-Curtis	0.831	0.850
	Unweighted Unifrac	1.796	**0.027***
	Weighted Unifrac	0.632	0.651
N1<br>Anxiety	Jaccard	1.047	0.285
	Bray-Curtis	0.972	0.518
	Unweighted Unifrac	1.777	**0.038***
	Weighted Unifrac	0.445	0.816
N2<br>Hostility	Jaccard	0.922	0.760
	Bray-Curtis	0.856	0.797
	Unweighted Unifrac	0.962	0.457
	Weighted Unifrac	0.550	0.727
N3<br>Depression	Jaccard	1.054	0.275
	Bray-Curtis	0.940	0.555
	Unweighted Unifrac	0.907	0.547
	Weighted Unifrac	0.801	0.509
N4<br>Self-consciousness	Jaccard	1.177	0.054
	Bray-Curtis	1.106	0.234
	Unweighted Unifrac	1.698	**0.038***
	Weighted Unifrac	0.719	0.547
N5<br>Impulsiveness	Jaccard	1.097	0.159
	Bray-Curtis	1.011	0.413
	Unweighted Unifrac	1.695	**0.037***
	Weighted Unifrac	0.453	0.799
N6<br>Vulnerability	Jaccard	1.187	0.067
	Bray-Curtis	0.967	0.557
	Unweighted Unifrac	2.120	**0.012***
	Weighted Unifrac	1.805	0.106

[a] Statistics were calculated using pairwise PERMANOVA with 999 permutations.
[*] $p < 0.05$
